# A promising preclinical test for DMD in dog models

**Published:** 2014-11

Duchenne muscular dystrophy (DMD) is a genetic disease that leads to muscle degeneration and loss of ambulation for which there are no available therapies. Preclinical research in animal models is thus crucial to develop new treatments, but predictive markers for disease progression and outcome, which would help assess the effects of a therapy, are currently limited. In this context, canine models of DMD – such as the Golden Retriever muscular dystrophy (GRMD) dog – are particularly valuable, as they share several similarities with the human disease, including a high inter-individual clinical heterogeneity. Here, Stéphane Blot, Gillian Butler-Browne and colleagues assessed motor dysfunction in GRMD dogs at 2 months of age, the time of clinical onset in this species. A few weeks later, one third of the animals displayed loss of ambulation, the most severe disease-related symptom. The authors found that the levels of circulating lymphocytes in the blood as well as the gait speed and stride frequency strongly correlated with the severity and progression of the disease in the animals. Notably, the same parameters have been shown to be predictive of loss of ambulation in DMD-affected individuals. Therefore, these three non-invasive biomarkers could immediately be used to screen experimental animals before testing specific therapeutic compounds. This approach not only will improve the reliability of preclinical trials performed on these large animals but will also hopefully speed up the discovery of new potential treatments for DMD. **Page 1253**

